# Effect of Reducing Agent on Solution Synthesis of Li_3_V_2_(PO_4_)_3_ Cathode Material for Lithium Ion Batteries

**DOI:** 10.3390/molecules25163746

**Published:** 2020-08-17

**Authors:** Ali Yaghtin, Seyyed Morteza Masoudpanah, Masood Hasheminiasari, Amirhossein Salehi, Dorsasadat Safanama, Chong Kim Ong, Stefan Adams, Mogalahalli V. Reddy

**Affiliations:** 1 Department of Physics, National University of Singapore, Singapore 117542, Singapore; ali.yaghtin93@gmail.com (A.Y.); amirhsalehi.23@gmail.com (A.S.); 2 School of Metallurgy and Materials Engineering, Iran University of Science and Technology (IUST), Narmak, Tehran 13114-16846, Iran; mhashemi@iust.ac.ir; 3 Department of Materials Science and Engineering, National University of Singapore, Singapore 117575, Singapore; mseds@nus.edu.sg (D.S.); mseasn@nus.edu.sg (S.A.); 4 Department of Mathematics, Xiamen University Malaysia, Jalan Sunsuria, Bandar Sunsuria, Sepang 43900, Selangor, Malaysia; ckong@xmu.edu.my; 5 Centre of Excellence in Transportation Electrification and Energy Storage (CETEES), Varennes, QC 1806, Canada

**Keywords:** Li_3_V_2_(PO_4_)_3_, solution synthesis method, reducing agent, electrochemical properties

## Abstract

In this study, Li_3_V_2_(PO_4_)_3_ (LVP) powders are prepared by a solution synthesis method. The effects of two reducing agents on crystal structure and morphology and electrochemical properties are investigated. Preliminary studies on reducing agents such as oxalic acid and citric acid, are used to reduce the vanadium (V) precursor. The oxalic acid-assisted synthesis induces smaller particles (30 nm) compared with the citric acid-assisted synthesis (70 nm). The LVP powders obtained by the oxalic acid exhibit a higher specific capacity (124 mAh g^−1^ at 1C) and better cycling performance (122 mAh g^−1^ following 50 cycles at 1C rate) than those for the citric acid. This is due to their higher electronic conductivity caused by carbon coating and downsizing the particles. The charge-discharge plateaus obtained from cyclic voltammetry are in good agreement with galvanostatic cycling profiles.

## 1. Introduction

Lithium ion batteries (LIBs) are promising electrochemical power sources that are used in portable electronics and hybrid electric vehicles because of their high energy density [1,2]. Among the cathode materials, vanadium-based oxides are one of the important electrode materials that are studied due to their variable V^3+,4+,5+^ oxidation state. Early work on a vanadium-based oxide, Li_x_V_2_O_5_, was conducted in 1979 by Dickeson et al. [3], and further details on the early history of lithium batteries have been summarized by Reddy et al. [4]. More recently, various studies has been carried out on vanadium-based cathodes with different crystal structures: triclinic [5,6,7,8,9,10], tetragonal [11], and orthorhombic [12,13]. Furthermore, Li_3_V_2_(PO_4_)_3_ (LVP) has been studied for its potential for practical applications in LIBs due to its unique properties such as thermal stability and high theoretical specific capacity [14,15,16]. The covalence P-O bonds and 3D solid framework of (PO_4_)^3−^ anions guarantee both good dynamic and thermal stabilities of LVP cathode [17]. The LVP has also a high theoretical specific capacity of 197 mAh g^−1^ and a moderate working voltage [18]. LVP has two crystal structures; rhombohedral and monoclinic structures, in which the monoclinic LVP shows a higher specific capacity due to the intercalation/de-intercalation of three Li ions per formula unit in stable phase transitions from Li_3_V_2_(PO_4_)_3_ to V_2_(PO_4_)_3_ [19]. The monoclinic LVP has specific capacities of 133 and 197 mAh g^−1^ as a function of applied electrochemical windows of 3.0–4.3 and 3.0–4.8 V vs. Li^+^/Li, on the basis of the de/intercalation of two and three Li^+^ ions, respectively [20]. However, the pristine LVP exhibits poor rate performance caused by its low electronic conductivities, which can be mitigated by downsizing, carbon coating, and aliovalent doping [21,22,23].

The wet-chemical synthesis of LVP is generally based on the reduction of cost-efficient V(V) precursors V_2_O_5_ and NH_4_VO_3_, to V(IV) species by organic reducing agents such as oxalic acid, citric acid, glucose, and sucrose [24,25,26]. The V(IV) is then reduced to V(III) at high temperatures during the calcination process for the formation of LVP. The organic reductants can function both as a chelating agent and a carbon source, affecting the physiochemical properties of obtained LVP powders [24]. No comparison of the role of reducing agent type on the electrochemical properties has been performed thus far. To clarify the comparison, the oxalic acid and citric acid were used as reducing agents. Furthermore, the solution method was selected for synthesis of LVP/C composite material due to factors such as simplicity, versatility, and time- and energy-efficiency [27,28,29]. The solution method is based on dissolving the metal precursors together with a suitable organic agent (such as CTAB) in water as a solvent. The precursor solution is then dried and decomposed at low temperatures (<350 °C). To obtain the final product, the dried gel is calcined at high temperatures (700–900 °C) [30]. The thermal decomposition rate, organic agents, calcination temperatures, and times, etc. are crucial factors in the powder characteristics.

In this work, the phase, morphology, and electrochemical properties of LVP powders were studied as a function of the type of reducing agent. The smaller particles and higher carbon coating of the LVP prepared by the oxalic acid led to higher capacity and cyclic stability, despite their lower specific surface area.

## 2. Experimental Procedure

### 2.1. Synthesis of Li_3_V_2_(PO_4_)_3_ Powders

V_2_O_5_ (Aldrich, Darmstadt, Germany), oxalic acid, H_2_C_2_O_4_, (Aldrich), citric acid, C_6_H_8_O_7_ (Aldrich), NH_4_H_2_PO_4_, and Li_2_CO_3_ were used as starting materials. V_2_O_5_, NH_4_H_2_PO_4_, Li_2_CO_3_ were in a molar ratio of 1:3:1.5. 0.4 mmol cetyltrimethylammonium bromide (CTAB; (C_16_H_33_)N(CH_3_)_3_]^+^ Br^−^) was successively dissolved into 50 mL of deionized water at 80 °C under vigorous stirring. The precursor solution was initially dried and then preheated at 350 °C for 20 min in a box furnace under air atmosphere. The powders obtained were finally calcined at 700 and 800 °C for 3 and 6 h under an argon atmosphere in a tube furnace (Carbolyte, Hope Valley, UK), with a heating rate of 3 °C/min.

### 2.2. Material Characterization Methods

Thermal analysis was performed using a STA 503 (BäHR, Hüllhorst, Germany) simultaneous thermal analyzer under air atmosphere at a heating rate of 5 °C min^−1^. The phases and structure of the LVP powders were analyzed using a D8 ADVANCE (Bruker, Kanagawa, Japan) X-ray powder diffraction (XRD) instrument with Cu Kα (λ = 1.54060 Å) radiation in the 2θ range of 10–60° at the scan rate of 0.04°/0.5 s. The morphology and microstructure of the powders were analyzed using a VEGA II (TESCAN, Brno, Czech Republic) scanning electron microscopy (SEM) device in the backscattered-electron mode at 15 kV and JEM-2010F (JEOL, Tokyo, Japan) transmission electron microscopy (TEM) apparatus at 200 kV. The textural properties of the composites were characterized using their N_2_ adsorption/desorption isotherms, which were obtained employing a Tristar 3000 (Micromeritics, Norcross, GA, USA) porosity analyzer. A P50C0R10 (Takram, Gilan, Iran) Raman spectroscope with a Nd:YAG laser was used to analyze the carbon coating. An X-ray photoelectron spectrometer (XPS) was used for detection of state of element using an AXIS ultra DLD spectrometer (Kratos Analytica, Manchester, UK) equipped with a monochromatic Al-Kα radiation. Further details of instrumentation are reported elsewhere [31]. The XRD, Raman, TGA, SEM, TEM, and XPS experiments were performed on the LVP/C samples calcined at 800 °C.

### 2.3. Electrochemical Characterization

The electrochemical properties were characterized in 2016 type coin cells. The LVP, polyvinylidene fluoride (PVDF), and Super-P carbon black in 70:15:15 wt.% ratio, respectively, were firstly mixed in *N*-methyl-2-pyrrolidone. The slurry was coated on aluminum foil and then dried in a vacuum oven overnight. The coated foil was cut into pieces of circular shape with a diameter of 16 mm. The working electrode had approximately 2.5 mg cm^−2^ of active material. The lithium metal as a counter/reference electrode, glass fiber as a separator, and 1M LiPF_6_ (EC and DMC *v/v* = 1:1) solution as the electrolyte were used to assemble a coin cell in an argon-filled glove box (MBraum, Bayern, Germany). Galvanostatic charge-discharge experiments (Bitrode, USA battery tester, St. Louis, MO, USA) and cyclic voltammetry (Solartron-1470, Cambridge, UK) were performed in the voltage range of 3.0 to 4.8 V. Electrochemical impedance spectroscopy (EIS) was carried out on a Solartron 1260A system. Further details of instrumentation are reported elsewhere [32].

## 3. Results and Discussion

**Figure 1:**
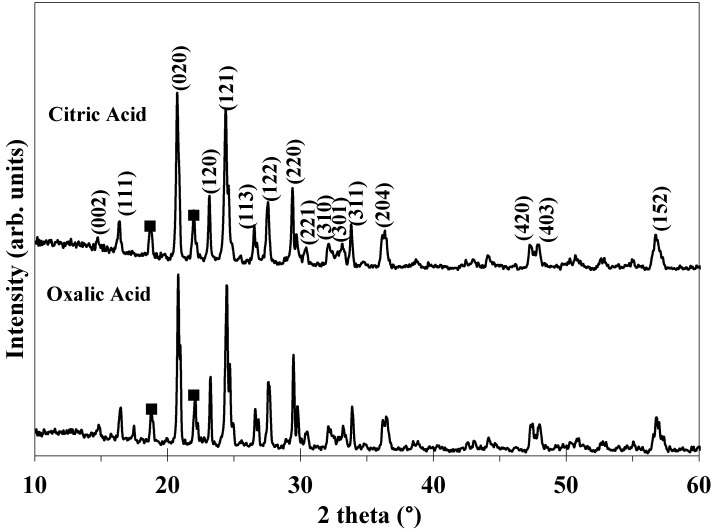
XRD patterns of the Li_3_V_2_P_3_O_12_ (LVP) compounds obtained by the oxalic and citric acids, Symbol ■ (Li_3_PO_4_).

Figure 1 shows the XRD patterns of the samples prepared by oxalic and citric acids as the reducing agents following calcination at 800 °C for 6 h. The indexed diffraction peaks are related to the monoclinic LVP structure with the space group of P21/n (PDF no. 01-072-7074). The calculated lattice parameters of LVP are a = 8.612, b = 8.636, and c = 12.049 Å for oxalic acid, and a = 8.658, b = 8.641, and c = 12.088 Å for citric acid. These are comparable with the reported values [33]. The oxalic acid leads to a lower lattice volume (888.12 Å^3^) in comparison with citric acid (900.01 Å^3^), possibly due to its higher crystallinity. Furthermore, there are no diffraction peaks of carbon in all the patterns due to its amorphous structure and/or the small thickness of the carbon layer on the LVP particles [34]. There are some diffraction peaks related to the Li_3_PO_4_ phase (PDF no. 00-015-0760) for both samples. The Li_3_PO_4_ phase is intermediate phase which reacts to form the final Li_3_V_2_(PO_4_)_3_ product. Therefore, the existence of impurity Li_3_PO_4_ phase can be attributed to the low calcination temperature of 800 °C. 

**Figure 2:**
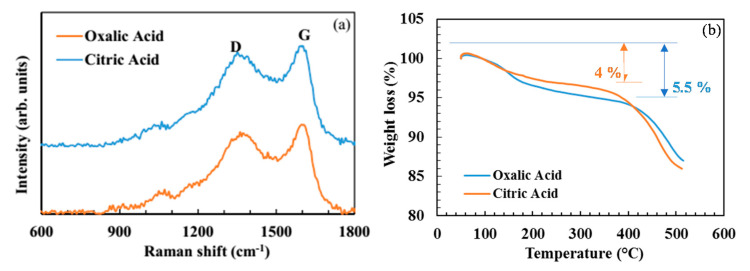
(**a**) Raman spectra and (**b**) TGA curves of the LVP compounds.

The Raman spectroscopy technique was used to identify the existence and nature of carbon in the synthesized LVP powders (Figure 2a). The two characteristic peaks at 1360 cm^−1^ (D-band) and 1560 cm^−1^ (G-band) are assigned to the sp^3^ and sp^2^ type carbon, respectively [35]. The absence of Raman peaks in the range of 500–1200 cm^−1^, corresponding to the vibrational mode of Li_3_V_2_(PO_4_)_3,_ is due to the coverage of particle surface by the amorphous carbon layer [36]. The carbon layer enhances the electrical conductivity without affecting the crystallinity of LVP [37]. Furthermore, the carbon layer improves the binding between the carbon additive and LVP in the electrodes. The intensity ratios of D to G band (I_D_/I_G_) as a criteria of graphitization are higher for oxalic acid (0.94) than for citric acid (0.89), indicating the predominance of sp^2^ type carbon domains. The carbon type has the higher contribution on the electrical conductivities. Carbon contents of the LVP particles determined from TGA curves (Figure 2b), are 5.5 wt.% and 4.5 wt.% for oxalic acid and citric acid, respectively. The higher carbon content by the oxalic acid may be due to its lower decomposition temperature. 

**Figure 3:**
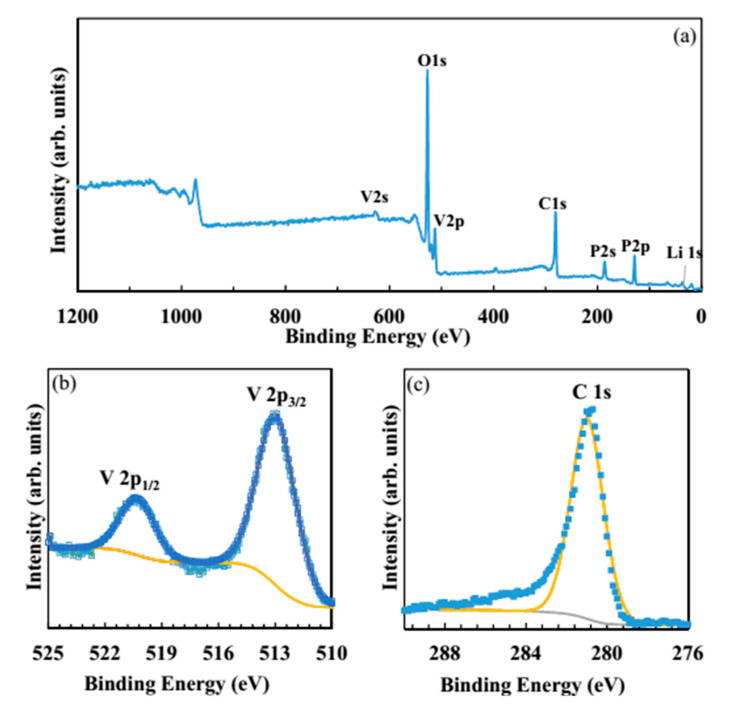
Wide range XPS spectrum showing (**a**), core level XPS spectra, (**b**) V2P, and (**c**) C 1s of the LVP compound prepared by the oxalic acid.

Figure 3a shows the overall X-ray photoelectron spectroscopy (XPS) examinations of the LVP powder that was synthesized by oxalic acid. The peaks of Li, V, O, P, and C elements are labeled. Figure 3b shows the binding energy of V2p_3/2_ and V2p_1/2_ at 511.6 and 519.6 eV, respectively. 

The observed binding energy values correspond to V^3+^ in LVP, suggesting the successful reduction of V^5+^ to V^3+^ [38]. The binding energy of a characteristic satellite peak of C1s at approximately 280.8 eV (Figure 3c) confirms the existence of carbon [39]. 

**Figure 4:**
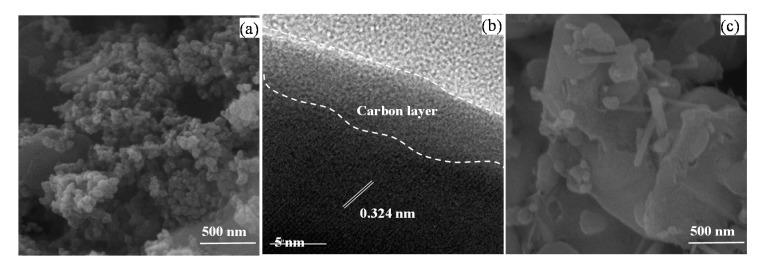
(**a**) SEM and (**b**) HRTEM images of the LVP compounds prepared by the oxalic acid and (**c**) SEM image for the citric acid.

**Figure 5:**
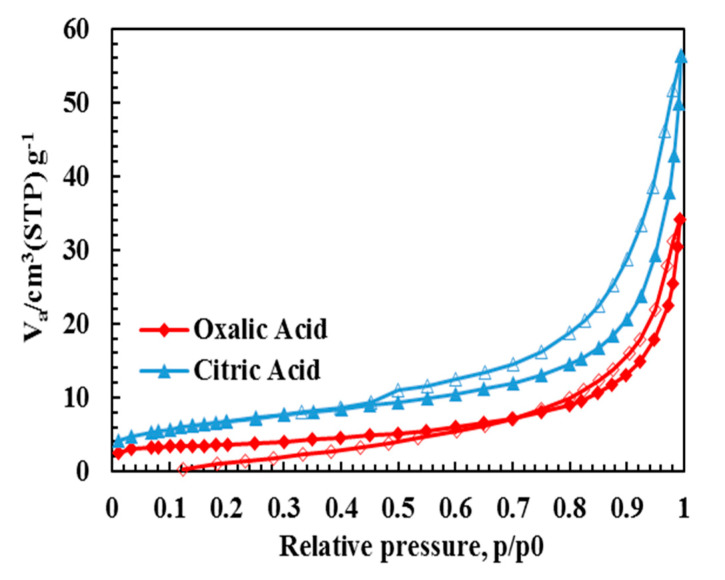
Adsorption (filled symbols)-desorption (open symbols) isotherms of the LVP powders.

SEM images of the LVP powders prepared by the oxalic acid and citric acid are compared in Figure 4a,c. When used as reducing agents, oxalic acid yields spherical nanoparticles (~30 nm), whereas citric acid yields bulky microstructures containing rod-like particles with a diameter of 50 nm and a length of 0.5 µm. The metal precursors are firstly decomposed to the corresponding oxides and phosphates [40,41,42]. The intermediate solid phases then react to form LVP during the calcination process [43]. The type and amount of metal precursors and organic agents control the decomposition and reaction rates, and therefore, the bulky microstructure can be attributed to the slower decomposition rate in the presence of citric acid caused by its carboxylate groups [44]. The HRTEM images (Figure 4b) clarify the crystal structure of nanoparticles and carbon layer on the particle surface. The interplanar spacing of 0.324 nm correspond to the (122) hkl plane, indicating high crystallinity. Furthermore, the average thickness of the carbon layer is approximately 5 nm. Figure 5 demonstrates that the N_2_ adsorption-desorption isotherms of the LVP powders are IV type with a typical H3 hysteresis. The LVP powders prepared by oxalic acid have a lower BET specific surface area (12 m^2^/g) than those of citric acid (24 m^2^/g). The higher pore volume of citric acid (0.08 cm^3^/g) than oxalic acid pore volume (0.05 cm^3^/g) can be attributed to the higher amounts of liberated gases. Furthermore, the pore sizes are mainly distributed at the mesopores range (1–20 nm) with the maximum at 6.5 nm for the oxalic acid, while the citric acid shows only the micropores (1–2 nm). The BET specific surface area and pore volume affects the electrochemical properties via tuning the ionic/electronic conduction pathways and mechanical response during cycling. Furthermore, the pores can partially overcome volume deformation during the Li^+^ de-/intercalation from/into the crystal structure [45].

**Figure 6:**
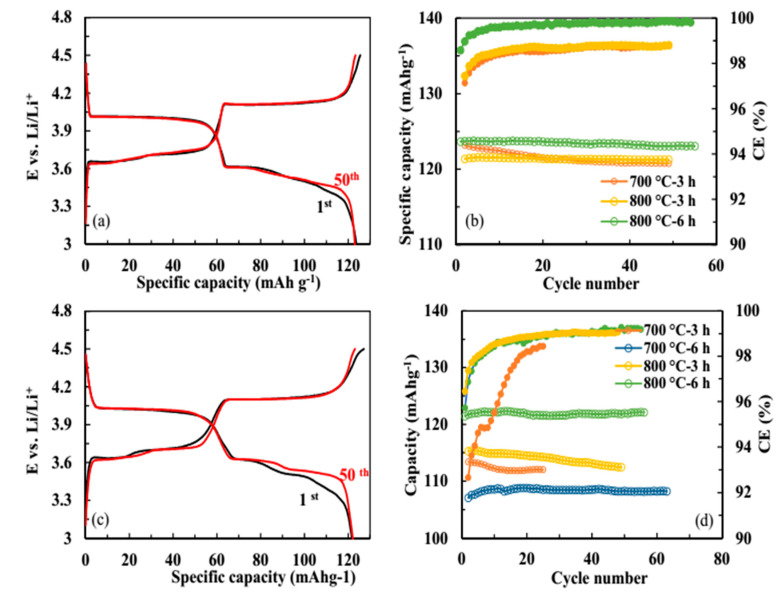
Initial and 50 th charge/discharge curves of the LVP compounds prepared by (**a**) oxalic acid and (**c**) citric acid and the cyclic performance of the LVP powders prepared by (**b**) oxalic acid and (**d**) citric acid, and potential (E) range of 3.0–4.3 V vs. Li.

Figure 6a,c compare the initial charge/discharge curves of LVP powders at 1C rate and in the range of 3.0–4.3 V. For the oxalic acid, the three charge plateaus are at ~3.63, 3.71, and 4.11 V and three discharge plateaus are at ~3.53, 3.62, and 4.00 V. The citric acid shows the plateaus at 3.62, 3.70, and 4.11 V for the charge process and 3.50, 3.61, and 4.00 V for the discharge process. The voltage plateaus of charge and discharge are related to the reversible phase transition processes, respectively [15,46,47,48,49]:

(1) $${\mathrm{Li}}_{3}V_{2}({\mathrm{PO}}_{4})3_{\;}⇌\;{\mathrm{Li}}_{2.5}V_{2}({\mathrm{PO}}_{4}{)}_{3}+0.5{\mathrm{Li}}^{+}+0.5e^{-}\;\;\;\;∼3.6\;V\;\mathrm{vs}.\;\mathrm{Li}/\mathrm{Li}$$

(2) $${\mathrm{Li}}_{2.5}V_{2}({\mathrm{PO}}_{4}{)}_{3}⇌\;{\mathrm{Li}}_{2}V_{2}({\mathrm{PO}}_{4}{)}_{3}+0.5{\mathrm{Li}}^{+}+0.5e^{-}\;\;\;\;∼3.7\;V\;\mathrm{vs}.\;\mathrm{Li}/\mathrm{Li}$$

(3) $${\mathrm{Li}}_{2}V_{2}({\mathrm{PO}}_{4}{)}_{3}\;⇌{\mathrm{Li}}_{1}V_{2}({\mathrm{PO}}_{4}{)}_{3}+1{\mathrm{Li}}^{+}+1e^{-}\;\;\;\;∼4.1\;V\;\mathrm{vs}.\;\mathrm{Li}/{\mathrm{Li}}^{+}$$

The voltage differences between the charge and discharge plateaus of LVP prepared by oxalic acid are smaller (~260 mV) than those of citric acid (~300 mV), confirming it has less polarization [50]. The initial discharge capacities of oxalic acid and citric acid at 1C are 124 and 123 mAh g^−1^, respectively, corresponding to 93.2% and 92.4% of the theoretical capacity (~133 mAh g^−1^) in the range of 3.0–4.3 V. The cycling performances of LVP powders prepared by the oxalic and citric acids at various calcination temperatures and times are summarized in Figure 6b,d. The oxalic acid shows higher and more stable discharge capacities than the citric acid. For the oxalic acid and calcination temperature of 800 °C, the specific discharge capacity of 124 mAh g^−1^ fades to 123 mAhg^−1^ after 50 cycles, corresponding to 99% capacity retention. The charge/discharge curves at 50th cycles also confirm the cyclic stability. However, the maximum specific discharge capacity of 122 mAhg^−1^, together with 99% capacity retention is obtained by the citric acid with the similar calcination process. The discharge capacities and capacity retention decrease with the decrease of calcination temperatures and times, due to the increase of amount of impurity phase and lower crystallinity. Furthermore, the Coulombic efficiencies of oxalic acid are higher than those of citric acid during 1C cycling, indicating the high reversibility of the material [51]. 

**Figure 7:**
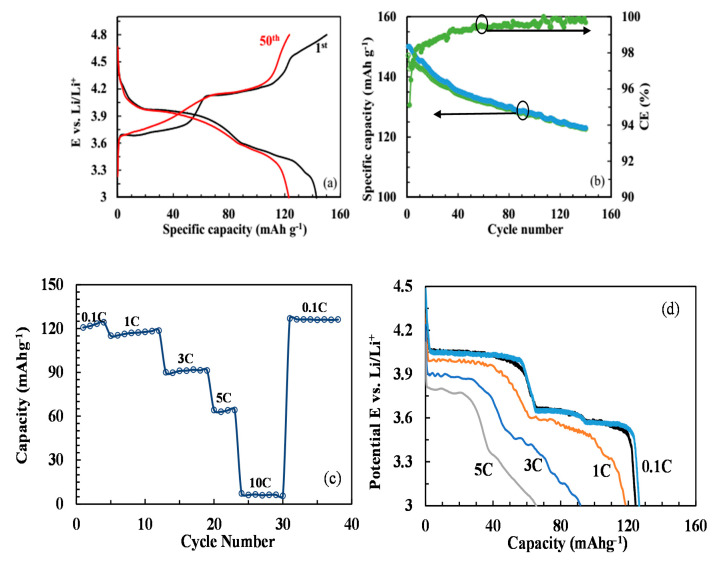
(**a**) Initial and 50 th charge/discharge curves, (**b**) cyclic performance, (**c**) rate performance and (**d**) charge/discharge curves of the LVP compounds prepared by the oxalic acid in the potential (E) range of 3.0–4.8 V Vs. Li.

Figure 7a,b show the charge/discharge curves and cycling performance of the LVP powders prepared by the oxalic acid in the voltage range of 3.0–4.8 V. The four plateaus at approximately 3.68, 3.74, 4.20, and 4.65 V vs. Li/Li^+^ in the charge curves (Figure 7a) correspond to a sequence of phase transitions of Li_x_V_2_(PO_4_)_3_, where x = 3.0, 2.5, 2.0, 1.0, and 0 [52,53,54]. The discharge curve, however, is S-shaped due to solid solution behavior, suggesting a single-phase reaction caused by a disordered lithium reinsertion [16]. With sufficient Li^+^ reinsertion, the two-phase behavior reappears for the reinsertion of the third lithium [55]. The capacity fading in the range of 3.0–4.8 V is more notable during long-term cycling than in the range of 3.0–4.3 V, which can be attributed to structural collapse during the phase transition from V_2_(PO_4_)_3_ to LiV_2_(PO_4_)_3_ in a solid solution regime at upper voltage of 4.8 V, as can be observed in the charge/discharge curves [56]. 

The rate performance of the LVP powders prepared by the oxalic acid and calcined at 800 °C is presented in Figure 7c,d. With the rapid current rate increase, the stable discharge capacity is obtained at each state. When the rate gradually increases from 0.1 to 1, 3, 5, and 10 C, the average discharge capacities decrease from 122.5 to 116.9, 90.9, 63.8, and 6.3 mAh g^−1^, respectively. The discharge capacity returns to 126.2 mAh g^−1^ when the current density returns to 0.1 C, indicating good rate capability of the synthesized LVP powders. This is explainable by the small spherical nanoparticles and carbon layer on the LVP particles, thereby improving the electronic conductivity [57]. Figure 7d shows that the voltage plateaus are degraded with the increased current densities. The charge/discharge curves reappear, however, by decreasing the current density to 0.1 C, indicating both structural and electrochemical stability at the higher current densities, in addition to the sufficient electron/ion conductivity of the synthesized LVP powders.

**Figure 8:**
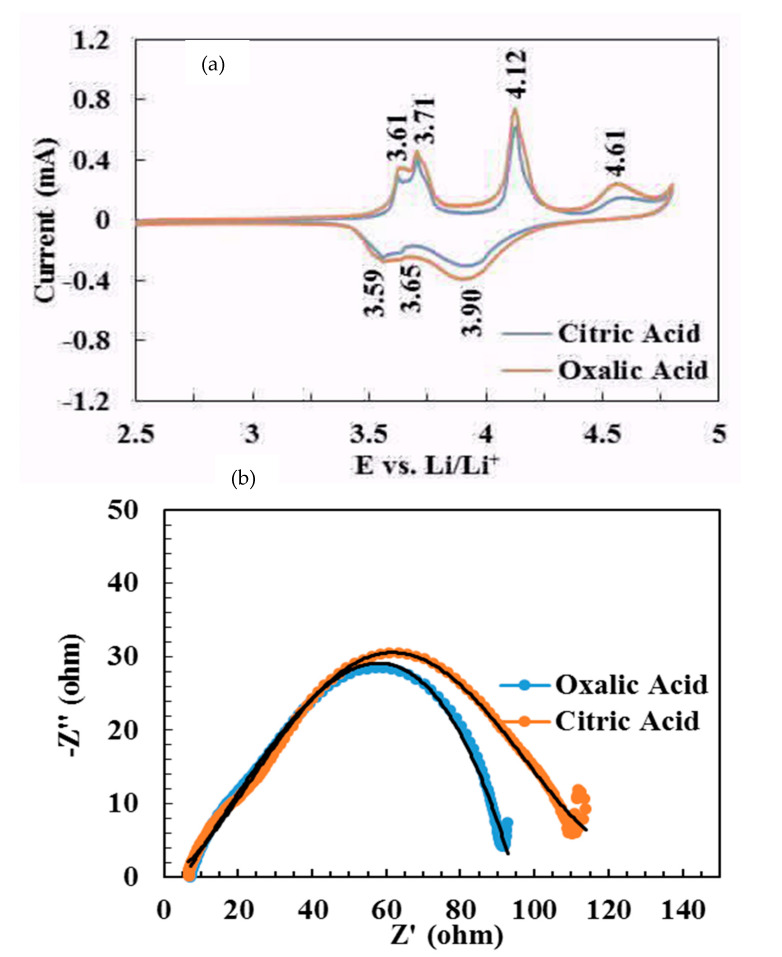
(**a**) Cyclic voltammograms of Li_3_V_2_P_3_O_12_ (LVP) prepared using two precursors. Scan rate, 0.1 mVs^−1^, for clarity second cycle are shown, Li metal as counter and reference electrode, and (**b**) Nyquist plots (Z′ vs. –Z″) of LVP electrodes.

Figure 8 compares the cyclic voltammetry curves of LVP powders at 0.1 mVs^−1^ in the voltage range of 3.0–4.8 V and the Nyquist plots. The LVP powders prepared by the oxalic acid have a high redox current and a large curve area. There are four peaks at 3.61, 3.71, 4.12, and 4.61 V in the charge process and three peaks at 3.59, 3.65, and 3.90 V in the discharge process, showing the reversible phase transitions during the Li-ion extraction/insertion [58]. The potential peaks at ~4.12 and 4.61 V appear as a single peak at ~ 3.90 V during the discharge process. This is attributed to the solid solution region from V_2_(PO_4_)_3_ to Li_2_V_2_(PO_4_)_3_ [59]. The results are in good agreement with the charge/discharge curves (Figure 7a and Figure 8b), showing the superior electrochemical properties of the LVP powders synthesized using the oxalic acid as the reducing agent. The impedance spectra consisted of a semicircle and a linear part at high and low frequency ranges, respectively. The diameter of the semicircle shows the combination of surface film (Rsf) +charge transfer resistance (Rct) and corresponding capacitance due to surface film (CPEsf) and double layer (CPEdl) which mainly affects the cathode impedance [6,60]. The smaller diameter of the semicircle for the oxalic acid (95 Ω) than the diameter of the semicircle for the citric acid (112 Ω) confirms its smaller R(sf+ct) resistance [61]. We also note Warburg region clearly noted when compared to our previous impedance studies of other cathode and anode materials [6,62,63,64,65], further careful studies on impedance studies at various voltages during charge and discharge cycle and cycle number are needed.

Table 1 compares the discharge capacity and retention of various Li_3_V_2_(PO_4_)_3_ cathode materials. It is worth to note that the Li_3_V_2_(PO_4_)_3_/C samples synthesized by the oxalic acid show the comparable discharge capacity and cycling performance in the voltage range of 3.0–4.3 V vs. Li^+^/Li.

## 4. Conclusions

The dependency of structural, microstructure, and electrochemical properties of Li_3_V_2_(PO_4_)_3_ powders were examined as a function of reducing agent and calcination temperatures (700 and 800 °C) and times (3 and 6 h). The smaller particle size obtained by means of the oxalic acid led to a lower charge-transfer resistance (95 Ω) compared with that for the citric acid (112 Ω. Following calcination at 800 °C for 6 h, the LVP powders synthesized by the oxalic acid showed the highest specific discharge capacity of 122.5 mAhg^−1^ at 1C and higher cycling performance (capacity retention of 99.8% following 55 cycles). With decreased calcination temperatures and times, the specific capacity faded during cycling and the Columbic efficiencies decreased due to the decreased crystallinity. The better electrochemical performance achieved by oxalic acid was mainly attributed to its smaller spherical nanoparticles. Further studies are needed on the applicability of this material with solid electrolyte for all solid State Batteries [66].

## Figures and Tables

**Table 1:** Summary of electrochemical properties of Li_3_V_2_(PO_4_)_3_/C cathode material.

Material	Synthesis Method	Capacity (mAh g^−1^, at 1C Rate	Retention (%)	Reference
Li_3_V_2_(PO_4_)_3_/RGO	Solvothermal	128, 1C	98	[18]
Li_3_V_2_(PO_4_)_3_/C	Solvothermal	112, 1C	97	[20]
Li_3_V_2_(PO_4_)_3_ in carbon matrix	Solvothermal	122, 1C	100	[21]
Li_3_V_2_(PO_4_)_3_/C	Microemulsion	130, 1C	97	[22]
Li_3_V_2_(PO_4_)_3_/C	Solution synthesis	130, 0.2C	93	[24]
Li_3_V_2_(PO_4_)_3_/C	Solvothermal	122, 1.5C	98	[26]
Li_3_V_2_(PO_4_)_3_/C	Freeze-drying	130, 0.1C	98	[27]
Li_3_V_2_(PO_4_)_3_/C	Microwave	130, 1C	99	[29]
Li_3_V_2_(PO_4_)_3_/C	Solution synthesis	124, 1C	98	This work
